# Association of osteopontin and cyclooxygenase-2 expression with breast cancer subtypes and their use as potential biomarkers

**DOI:** 10.3892/ol.2013.1600

**Published:** 2013-10-01

**Authors:** DHANASHRI THORAT, ASUTOSH SAHU, REETI BEHERA, KIRTI LOHITE, SANJAY DESHMUKH, ANUPAMA MANE, SWAPNIL KARNIK, SUHASCHANDRA DOKE, GOPAL C. KUNDU

**Affiliations:** 1 Department of Tumor Biology, Angiogenesis and Nanomedicine, National Centre for Cell Science, Pune, Maharashtra 411007, India; 2 Grant Medical Foundation, Ruby Hall Clinic, Pune, Maharashtra 411001, India; 3 Institute for Advanced Computing and Life Sciences, Pune, Maharashtra 411004, India

**Keywords:** invasive ductal carcinoma, breast cancer subtypes, TNM staging, estrogen receptor, progesterone receptor, human epidermal growth factor receptor type 2

## Abstract

Breast cancer is one of the most common malignant tumors among females worldwide and remains a leading cause of cancer-related mortality. Due to the heterogeneous clinical nature of breast cancer, it is necessary to identify new biomarkers that are associated with tumor growth, angiogenesis and metastasis. Osteopontin (OPN) and cyclooxygenase-2 (COX-2) are known to be overexpressed in invasive breast cancer and their overexpression is associated with aggressive histological and clinical features. The present study assessed OPN and COX-2 expression in various subtypes of breast cancer. The expression of OPN and COX-2 was analyzed using immunohistochemistry (IHC) in a cohort of 67 invasive ductal breast carcinoma patients. The statistical analysis was performed using standard statistical software SPSS version 18.0. The associations between OPN and COX-2 and the human epidermal growth factor receptor type 2 (HER2)-overexpressing and non-HER2-overexpressing subtypes were evaluated using the Mann-Whitney U test. The mean OPN level was significantly higher in the HER2-overexpressing subtype compared with the non-HER2-overexpressing subtype. Furthermore, the mean COX-2 expression levels were higher in the HER2-overexpressing subtype compared with the luminal A, luminal B or triple-negative groups. It is well known that carcinomas overexpressing HER2/neu have a worse prognosis than luminal tumors. Hence, it may be hypothesized that an elevated expression of OPN and COX-2 in a HER2-overexpressing subtype may contribute to a more aggressive behavior and be used as diagnostic and prognostic markers in breast cancer.

## Introduction

Breast cancer is one of the most common malignant tumors among females worldwide. Although mortality rates are decreasing due to combined therapy, breast cancer remains a leading cause of cancer-related mortality in females. In India, breast cancer has overtaken cervical cancer, which was the most common cancer a decade ago (1).

The studies from the National Cancer Institute (NCI; National Institutes of Health) indicated that 226,870 females would be diagnosed with breast cancer and 39,510 would succumb to this disease during 2012. The data from the Indian population based cancer registry (PBCR; 2006–2008; Indian Council of Medical Research) suggest that breast cancer accounts for 28–35% of all cancers in females within the major cities of India. A total of 130 million Indian females are expected to live beyond the menopause into old age by 2015 (2). While the natural age of menopause in developed countries is 51 years, in India the mean age is ~45 years (3). As the breast cancer risk is high among post-menopausal women, it is predicted that breast cancer may be a major cause of mortality in India in the next few decades. Therefore, it is strongly argued that India should adopt screening strategies for the early diagnosis of cancer, as it is usually associated with an improved clinical outcome and the increased overall survival of patients.

The expression profiles of estrogen receptor (ER), progesterone receptor (PR) and human epidermal growth factor receptor (HER2)/neu have been used for predicting the outcome and response to the therapy of breast cancer for a number of years. However, the assessment of these clinical and pathological features is not sufficient to fully capture the heterogeneous clinical course of breast cancer, making it necessary to identify new biomarkers that are associated with growth, angiogenesis and metastases.

Osteopontin (OPN), a secreted, non-collagenous, extracellular matrix protein that belongs to the small integrin-binding ligand N-linked glycoprotein (SIBLING) family, plays a significant role in determining the oncogenic potential of various cancers and is recognized as a key marker in the processes of tumorigenicity and metastasis (4). OPN is involved in normal tissue remodeling processes, including bone resorption, wound healing and tissue injuries, in addition to restenosis, atherosclerosis, tumorigenesis and autoimmune diseases (5,6). OPN has been shown to play a significant role in tumor invasion and metastasis in breast, lung, prostate and colon cancers. Due to its known tumor-associated biological functions, OPN appears to have the potential to aid in the identification of high-risk tumors. Therefore, the detection of OPN expression levels in breast cancer patients may be useful in establishing its role as a diagnostic marker (7,8).

In breast cancer, high OPN levels in the tumor tissue are associated with a poor prognosis and disease progression (9). OPN acts as a clinical prognostic marker and is a key player in the six hallmarks of cancer that include self-sufficiency in growth signals, insensitivity to growth-inhibitory signals, evasion of apoptosis, limitless replicative potential, sustained angiogenesis and tissue invasion and metastasis in the model of breast cancer (10). A previous study has shown that a higher fraction of breast cancer is identified by the detection of OPN-c compared with ER, PR or HER-2 and that OPN-c may be used as a diagnostic and prognostic marker. This may be particularly useful as ER and PR are considered to be weak prognostic markers (11–14).

The cyclooxygenases (COXs) are a family of myeloperoxidases that are located at the luminal side of the endoplasmic reticulum and nuclear membrane. COXs catalyze the rate-limiting step of prostaglandin biosynthesis from arachidonic acid. To date, three COX isoforms have been identified, COX-1, COX-2 and COX-3. COX-1 is constitutively expressed in various tissues and plays a role in tissue homeostasis (15).

COX-2 is an inducible isoform, which is overexpressed during inflammation, and is regulated by growth factors and various cytokines, including IL1β, IL6 or tumor necrosis factor (TNF)-α (16). COX-3 has been identified as a splice variant of COX-1 and is present mainly in the brain and spinal cord, but its role is not clearly understood (17,18). There are various studies with regard to COX-2 overexpression in invasive breast cancer and ductal carcinoma *in situ,* and the overexpression of COX-2 has been identified to be associated with aggressive histological and clinical features (19–26).

However, to date, there are no data with regard to OPN and COX-2 overexpression and their correlation with various subtypes of breast cancer. The present study was designed to provide an improved definition of the combined effect of OPN and COX-2 overexpression in the progression of breast cancer, and to analyze the correlation between the expression pattern and various subtypes of breast cancer.

## Materials and methods

### Study population

Approval for the present study was obtained from the ethical committee of Ruby Hall Clinic (Pune, Maharashtra, India). Formalin-fixed paraffin-embedded breast tumor specimens were obtained from the Department of Histopathology, Ruby Hall Clinic. Records of 375 breast cancer patients treated between 2006 and 2010 were obtained. Patients were excluded from the study if they were male, had a metastatic disease at the time of diagnosis or were administered any kind of chemotherapy or radiation therapy prior to the surgery. Patients with only carcinoma *in situ* or with bilateral breast cancer were also excluded from this study. The records of the patients were retrieved and the clinical data, histopathological records and treatment information were all reviewed. The tumor grades of the invasive carcinomas were classified according to the Scarff-Bloom-Richardson system (27). The presence of lymph node metastases was reviewed for each patient. The tumor-node-metastasis (TNM) stage was determined according to the American Joint Committee on Cancer’s Cancer Staging Manual (28). The carcinomas were histologically divided into ductal, lobular and other tumors. The age of menopause was decided according to the mean age of menopause in India (3).

### Antibodies and reagents

Mouse monoclonal anti-OPN and goat polyclonal anti-COX-2 antibodies and horseradish peroxidase (HRP)-conjugated IgG were purchased from Santa Cruz Biotechnology (Santa Cruz, CA, USA). The Super Sensitive Polymer HRP Immunohistochemistry (IHC) Detection System was purchased from Biogenex (QD 400,60K; Life Sciences Pvt Ltd., Hyderabad, AP, India).

### IHC staining

The specimens that were embedded in paraffin blocks were cut into 5-μm sections on poly-L-lysine coated slides. IHC was performed using the IHC detection system (Biogenex). Briefly, the sections were deparaffinized and subjected to antigen heat retrieval in a citrate buffer (pH 6.0) at 90°C for 30 min. Endogenous peroxidase activity and non-specific binding were blocked by incubation with a peroxide block and a power block, respectively, using an IHC kit (BioGenex, Life Sciences Pvt. Ltd.). The slides were then incubated sequentially with primary antibodies overnight at 4°C and then with their respective secondary antibodies for 1 h at room temperature. Diaminobenzidine hydrochloride (DAB) was used as chromogen. Subsequently, the sections were counterstained with hematoxylin and mounted using DPX mounting media.

### IHC scoring

IHC scoring was performed as previously described. Briefly, the tumor staining was semi-quantitatively examined by an oncopathologist using a double-blinded procedure with the Allred 8-unit IHC scoring system. The cytoplasmic staining of OPN and COX-2 was scored based on two parameters, staining intensity and positivity (29). Overall staining (staining index) was calculated by the sum of the intensity (I) and positivity (P); I + P = 0–8. A staining index of more than four was defined as high expression, while less than four was defined as low expression.

### Statistical analysis

The statistical analysis was performed using standard statistical software SPSS version 18.0 (SPSS, Inc., Chicago, IL, USA). The differences in the clinicopathological characteristics, including the TNM stage, tumor grade and lymph node status, between the HER2-overexpressing and non-HER2-overexpressing subtypes of breast cancer were calculated using the χ^2^ and Fisher’s exact tests. The associations between OPN and the HER2-overexpressing and non-HER2-overexpressing subtypes were evaluated using the Mann-Whitney U Test. The Kruskal-Wallis test was used to evaluate the association between the mean score of OPN and the TNM stage, histological subtype and tumor grade of the patients. All the statistical tests were two-sided. P<0.05 was considered to indicate a statistically significant difference.

## Results

### Association between tumor subtypes of breast carcinomas and clinicopathological parameters

Of the 375 breast cancer patients, 287 patients had complete information on the ER, PR and HER2 statuses. The baseline characteristics of the subjects, including the tumor subtypes are presented in Table I. Of these 287 subjects, 87 (30.3%) were of the luminal A subtype, 110 (38.3%) were of the luminal B subtype, 46 (16.0%) were of the HER2-overexpressing subtype and 44 (15.3%) were of the triple negative subtype. The median age of the patients was 54 years (SD, 12; range, 23–83 years; Table I).

Patients in the HER2-overexpressing and triple negative groups were more likely to have a higher grade of tumor, with 32% of these two groups being grade 3 at the time of diagnosis compared with 14% of the luminal cohort (P=0.000; Table I). There were no grade 1 cases in either the HER2-overexpressing or triple negative subtypes. The triple negative subtype was more frequently associated with a higher T-stage compared with the non-triple negative subtypes (Tables I and II; Fig, 1). The other tumor subtypes did not significantly correlate with the tumor grade, stage or lymph node status.

### Correlation between OPN expression and the tumor subtypes and clinicopathological features

**Figure 2:**
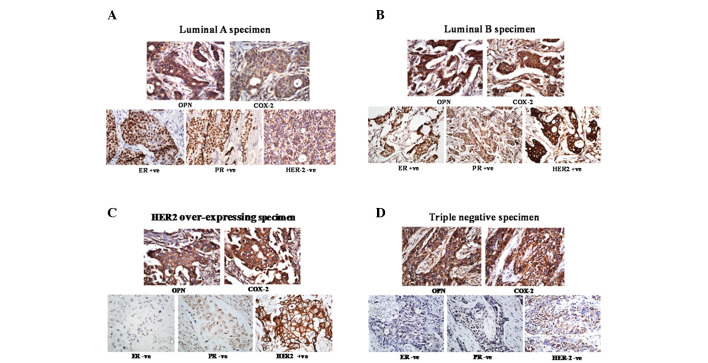
Representative images of osteopontin (OPN) and cyclooxygenase-2 (COX-2) expression in the breast cancer subtypes, including (A) luminal A (B) luminal B, (C) HER2-overexpressing and (D) triple negative subtypes. HER2, human epidermal growth factor receptor type 2. DAB staining; magnification, ×40.

**Figure 3:**
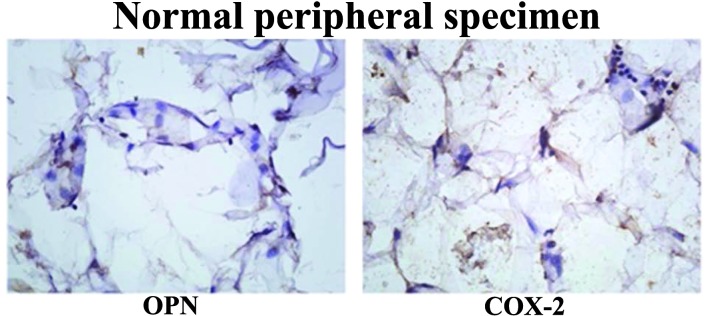
OPN and COX-2 expression in peripheral normal breast tissue. OPN, osteopontin; COX-2, cyclooxygenase-2.

The expression of OPN in the 67 primary tumors (18 luminal A, 17 luminal B, 15 HER2-overexpressing and 17 triple negative tumors) was analyzed using IHC. The representative images are shown in Fig. 2. IHC scoring was performed as described in the materials and methods section. The results revealed that the mean OPN level was significantly higher in the HER2-overexpressing subtype than in the non-HER2-overexpressing subtypes (P=0.043; Table III). However there was no correlation between OPN expression and the triple negative subtype of breast cancer. Furthermore, OPN expression did not correlate with any of the clinicopathological features that were evaluated, including age, pathological grading, histological subtype, tumor stage and lymph node metastasis (Table III). The expression of OPN and COX-2 was examined in the peripheral normal specimens and negligible expression of these proteins was identified compared with the tumor specimens of the multiple subtypes (Fig. 3). Furthermore, fibroadenoma specimens were analyzed and the results indicated that there was weak expression of OPN and COX-2 (data not shown).

### Association of COX-2 expression with tumor subtypes and clinicopathological features

The expression of COX-2 in the 66 primary tumors (18 luminal A, 17 luminal B, 15 HER2-overexpressing and 16 triple negative tumors) was analyzed by IHC and it revealed no significant correlation between COX-2 expression and the clinicopathological features. The mean COX-2 level was higher in the HER2-overexpressing subtype than in the luminal A, luminal B or triple negative groups. However, the correlation was not identified to be statistically significant when the tumor subtypes were divided into HER2-overexpressing and non-HER2-overexpressing groups (P=0.101; Table III).

## Discussion

A total of 1,638,910 new cancer cases and 577,190 mortalities from cancer were predicted to occur in the USA in 2012, which accounted for ~23% of the total mortalities (30). However, over the last few decades, there have been significant advances in breast cancer management, leading to the early detection of the disease and the development of more effective treatment modalities, which has resulted in a significant decline in breast cancer mortalities and improved outcomes of females with the disease (31,32). Breast cancer is no longer considered to be a single disease, but rather a multifaceted disease comprised of distinct biological subtypes and a diverse natural history, thus presenting a varied spectrum of clinical, pathological and molecular features with various prognostic and therapeutic implications.

A previous study showed that the new molecular classification of breast cancer is of significant prognostic value (33). The subtyping of breast cancer using microarrays is an efficient method to perform a molecular classification. However, the majority of the archived clinical specimens are not amenable to such an analysis. These assays are also limited to research laboratories and therefore are not advantageous for clinical practice. The IHC-based classification systems remain of use in clinical practice, particularly when fresh tissue is not available, and has been shown to correlate well with the intrinsic classification using gene expression by microarrays: ER/PR^+^ and HER2^−^ with luminal A; ER/PR^+^ and HER2^+^ with luminal B; ER^−^, PR^−^ and HER2^+^ with the HER2-overexpressing group; and ER^−^, PR^−^ and HER2^−^ with triple negative breast cancer (34–39). Early relapse and mortality were more frequent among the HER2-overexpressing and triple negative subtypes. Several studies have shown a trend towards a poor outcome for patients with cancer belonging to these groups (40–42).

The data of the present study indicated that the HER2-overexpressing and triple negative subtypes were associated with higher nuclear and histological grades of tumor, while only the triple negative subtype was associated with a higher pathological T-stage. The present study aimed to establish the level of expression and clinical significance of OPN and COX-2 in patients presenting with various subtypes of breast cancer. It was observed that the HER2-overexpressing subtype of breast cancer was significantly associated with OPN overexpression. The mean OPN and COX-2 levels were significantly higher in the HER2-overexpressing breast cancer group. The HER2 oncoprotein is a transmembrane receptor, belonging to the epidermal growth factor receptor family, with tyrosine kinase activity, resulting in intracellular signaling and the activation of genes that are involved in cell growth, which is associated with shortened survival rates, enhanced aggressiveness and a poor prognosis. Therefore, abnormal OPN and COX-2 expression may contribute to the aggressive behavior and poor prognosis in patients with the HER2-overexpressing subtype. Additional prospective and molecular level studies are required for an improved understanding of the role of OPN and COX-2 in the HER2-overexpressing subtype.

## Figures and Tables

**Figure 1:**
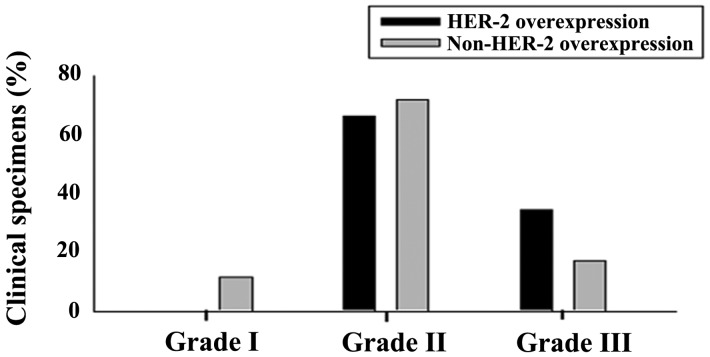
Representation of HER2-overexpressing and non-HER2-overexpressing specimens across tumor grades.

**Table I:** Differences in the clinicopathological characteristics between various subtypes of breast cancer.

		Subtype, n				
			
Characteristics	n	Luminal A	Luminal B	HER2-overexpressing	Triple negative	P-value
Age at diagnosis, years						
≤ 45	64	16	29	4	15	
>45	223	71	81	42	29	0.016
T Stage						
1	45	19	15	7	4	
2	124	33	42	24	25	
3	19	2	7	3	7	
4	10	2	5	3	0	0.130
Tumor grade						
1	26	17	9	0	0	
2	190	58	75	27	30	
3	53	7	19	14	13	0.000

HER2, human epidermal growth factor receptor type 2.

**Table II:** Tumor grade representation in the HER2-overexpressing and non-HER2-overexpressing subtypes of breast cancer.

Type	Grade I, % (n)	Grade II, % (n)	Grade III, % (n)	Total no. of specimens
HER2-overexpressing (Score, 3+)	0	65.85 (27)	34.14 (14)	41
Non-HER2-overexpressing (Luminal A, B and triple negative)	11.4 (26)	71.49 (163)	17.10 (39)	228

HER2, human epidermal growth factor receptor type 2.

**Table III:** Correlation of OPN and COX-2 with the tumor subtypes and clinicopathological parameters.

	OPN expression			COX-2 expression		
		
Clinicopathological features	n	Score^a^	P-value	n	Score	P-value
HER2 overexpression	15	6.20±0.94		15	5.80±1.20	
Non-HER2 overexpression	52	4.56±2.68	0.043	51	4.63±2.20	0.101
Tumor stage						
1	12	5.92±2.10		12	5.42±1.50	
2	46	4.59±2.58		45	4.64±2.32	
3	6	6.00±1.41		6	5.67±1.03	
4	2	3.00±4.24	0.261	2	5.00±1.41	0.898
Tumor grade						
1	4	3.25±3.77		4	3.25±3.77	
2	47	5.02±2.49		46	4.87±1.98	
3	15	4.93±2.15	0.455	15	5.33±1.79	0.708
Nodal status						
−	32	4.84±2.78		32	4.53±2.44	
+	32	4.87±2.29	0.432	31	5.23±1.68	0.566

a Scores obtained using Allred 8 unit IHC scoring system from 0 to 8; data are presented as the mean ± standard deviation.

OPN, osteopontin; COX-2, cyclooxygenase-2; HER2, human epidermal growth factor receptor type 2.
